# Mesoporous Silica Supported Pd-MnO_x_ Catalysts with Excellent Catalytic Activity in Room-Temperature Formic Acid Decomposition

**DOI:** 10.1038/srep33502

**Published:** 2016-09-26

**Authors:** Min-Ho Jin, Duckkyu Oh, Ju-Hyoung Park, Chun-Boo Lee, Sung-Wook Lee, Jong-Soo Park, Kwan-Young Lee, Dong-Wook Lee

**Affiliations:** 1 Advanced Materials and Devices Laboratory, Korea Institute of Energy Research (KIER) 152 Gajeongro, Yuseong, Daejeon 305-343, Republic of Korea; 2 Department of Chemical and Biological Engineering, Korea University Sungbuk-gu, Seoul 136-701, Republic of Korea; 3 Clean Fuel Laboratory, Korea Institute of Energy Research (KIER) 152 Gajeongro, Yuseong, Daejeon 305-343, Republic of Korea

## Abstract

For the application of formic acid as a liquid organic hydrogen carrier, development of efficient catalysts for dehydrogenation of formic acid is a challenging topic, and most studies have so far focused on the composition of metals and supports, the size effect of metal nanoparticles, and surface chemistry of supports. Another influential factor is highly desired to overcome the current limitation of heterogeneous catalysis for formic acid decomposition. Here, we first investigated the effect of support pore structure on formic acid decomposition performance at room temperature by using mesoporous silica materials with different pore structures such as KIE-6, MCM-41, and SBA-15, and achieved the excellent catalytic activity (TOF: 593 h^−1^) by only controlling the pore structure of mesoporous silica supports. In addition, we demonstrated that 3D interconnected pore structure of mesoporous silica supports is more favorable to the mass transfer than 2D cylindrical mesopore structure, and the better mass transfer provides higher catalytic activity in formic acid decomposition. If the pore morphology of catalytic supports such as 3D wormhole or 2D cylinder is identical, large pore size combined with high pore volume is a crucial factor to achieve high catalytic performance.

Hydrogen has attracted an increasing attention as a candidate for clean energy source when combined with polymer electrolyte membrane fuel cell (PEMFC) technology, and has the potential to play a very significant role in power generation system in the future^1^,^2^. One of the important challenging topics for such a hydrogen economy is to develop hydrogen storage materials. Formic acid (HCOOH) as a liquid organic hydrogen carrier (LOHC) is one of the most promising candidate for hydrogen storage materials, especially for portable applications, because its volumetric and gravimetric energy density are 53 g/L and 4.3 wt%, and they are relatively nontoxic, noncorrosive, and inflammable, which are crucial factors for portable devices. Formic acid can decompose in two different pathways, through either dehydrogenation reaction Eq (1) or dehydration reaction Eq (2)^3^.


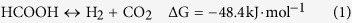



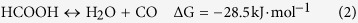


For the application of formic acid as a LOHC for an energy source for fuel cells, the dehydrogenation of formic acid is highly desired, and the catalytic selectivity for dehydrogenation and dehydration depends on the catalytic surface, temperature, pH value in reaction system, formic acid concentration and so on ref. 4. Recently, there have been numerous reports on excellent homogeneous catalysts for the formic acid dehydrogenation^4^,^5^,^6^,^7^,^8^. However, the limitation of homogeneous catalysts such as the difficulty of separation and recycle spontaneously provoked the study on heterogeneous catalysts for formic acid decomposition^9^. Since then, there have been intensive studies on development of efficient heterogeneous catalysts using various supports and metals so that their catalytic activity exceeds or is comparable to that of homogeneous catalysts^10^,^11^,^12^,^13^,^14^,^15^,^16^,^17^,^18^,^19^,^20^,^21^,^22^,^23^,^24^,^25^,^26^,^27^,^28^,^29^,^30^,^31^,^32^,^33^.

Tedsree *et al*.^9^ reported that Ag@Pd core-shell nanoparticles gave much better performance for formic acid decomposition at room-temperature than monometallic Pd nanoparticles. Mori *et al*.^34^ investigated FA decomposition of Pd and Pd-Ag nanoparticles within a basic resin with N(CH_3_)_2_ functional groups. They demonstrated that the weak basicity of resin and the small particle size of Pd are important factors in achieving good performances for formic acid decomposition at room-temperature. Qi-Long Zhu *et al*.^35^ synthesized well-dispersed ultrafine Pd nanoparticles supported on nanoporous carbon MSC-30 by using a sodium hydroxide-assisted reduction approach, and used them as a formic acid decomposition catalysts. Pd/MSC-30 catalyst showed a highly efficient and complete hydrogen generation in a formic acid-sodium formate system, providing TOF value as high as 750 h^−1^ at room-temperature. In another report, Fu-Zhan Song *et al*.^36^ prepared sub-2 nm Pd nanoparticles deposited on diamine-alkalized reduced graphene oxide. The catalysts gave excellent TOF of 3810 h^−1^ in a formic acid-sodium formate system at 323 K. In case of using additives such as sodium formate, the catalytic activity for heterogeneous catalysts was comparable to that for the most active homogeneous catalysts.

However, in case of additive-free formic acid decomposition, the catalytic activity of heterogeneous catalysts is still lower than that for formic acid-sodium formate system. Zhi-Li Wang *et al*.^37^ investigated additive-free formic acid decomposition using CoAuPd/C catalyst at room-temperature. The TOF and final conversion for Co_0.30_Au_0.35_Pd_0.35_/C catalysts were 80 h^−1^ and 91%, respectively. In our previous work, we synthesized novel mesoporous silica (KIE-6) by means of waste-glycerol templating, and utilized KIE-6 as a catalyst support for additive-free formic acid decomposition reaction. The Pd-MnO_x_ catalysts supports on NH_2_-functionalized KIE-6 showed the excellent catalytic activity (TOF: 540.6 h^−1^) for additive-free formic acid decomposition at room temperature^38^.

Most studies on catalysts for formic acid decomposition with or without additives have so far focused on the composition of metals and supports, the size effect of metal nanoparticles, and surface chemistry of supports. Another influential factor for formic acid decomposition performance is highly desired to overcome the current limitation of heterogeneous catalysis for formic acid decomposition. In this study, we investigated the effect of support pore size on formic acid decomposition performance without additives at room temperature, and achieved the excellent catalytic activity by controlling the pore size of catalyst supports.

## Results

### Glycerol-templated mesoporous silica (KIE-6) as a catalytic support for formic acid decomposition

**Figure 1:**
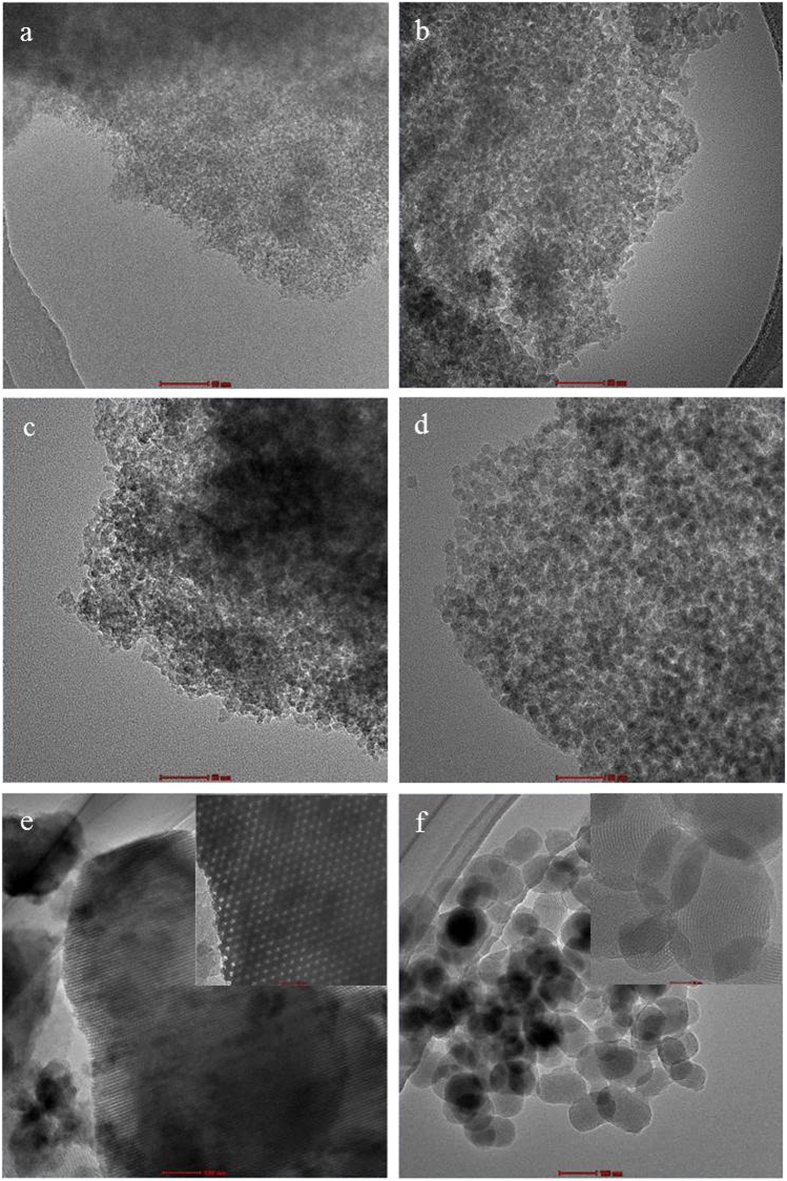
TEM images of mesoporous silica supports. (**a**) KIE-6-a, scale bar: 50 nm. (**b**) KIE-6-b, scale bar: 50 nm. (**c**) KIE-6-c, scale bar: 50 nm. (**d**) KIE-6-d, scale bar: 50 nm. (**e**) SBA-15, scale bar: 100 nm. (**f**) MCM-41, scale bar: 100 nm.

**Figure 2:**
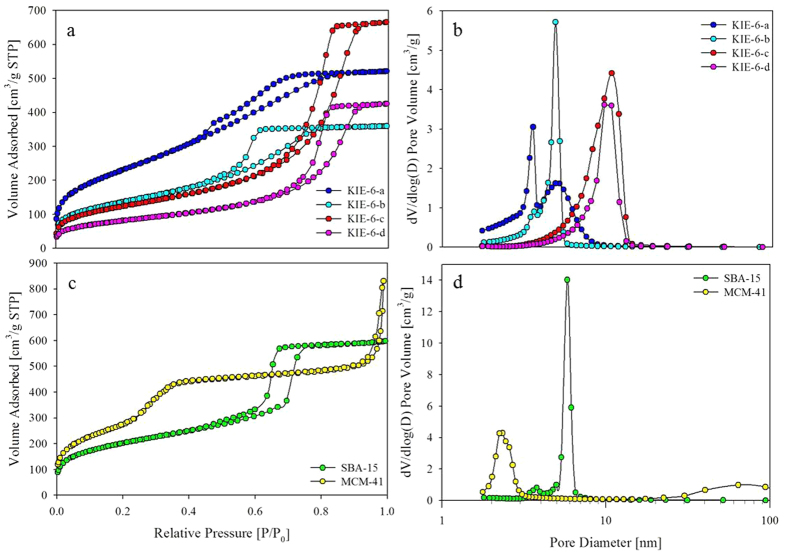
Nitrogen sorption results of KIE-6, SBA-15 and MCM-41. (**a,b**) KIE-6-a, -b, -c, -d. (**c,d**) SBA-15 and MCM-4. (**a,c**) Isotherms. (**b,d**) BJH desorption pore size distributions.

Previously, we reported glycerol-templated mesoporous silica (KIE-6) with controlled pore size and pore wall thickness^38^. To investigate the effect of pore structure of supports on catalytic activity, easy control of support pore structure is an essential prerequisite. Thus we used mesoporous silica KIE-6 as a catalytic support for formic acid decomposition, because the pore properties of KIE-6 such as pore size, pore volume, and surface area can be readily controlled by changing the concentration of glycerol template and silica nanoparticle size. In addition, it is well-known that silica supports is more efficient in formic acid decomposition than other oxides and their surface modification is easy. Mesoporous silica KIE-6 was prepared by using glycerol and silica nanospheres as a templating agent and silica source. The drying of mixture solution of glycerol and colloidal silica sol leads to formation of glycerol-silica nanosphere (G-SN) nanocomposites via hydrogen bonding interaction. Afterward carbonization of G-SN nanocomposites gives rise to formation of carbon-silica nanosphere (C-SN) nanocomposites combined with consolidation of mesostructured silica nanospheres via condensation between adjacent silica nanospheres. Finally mesoporous silica KIE-6 is synthesized by elimination of carbon from C-SN nanocomposites via calcination in air. Compared to surfactant-templated mesoporous silica such as MCM-41, SBA-15 and so on, the pore size of KIE-6 can be more easily controlled by simply changing glycerol concentration or silica nanosphere size. In this study, we synthesized KIE-6-a, -b, -c, and -d with different pore properties. The synthesis conditions of KIE-6 were summarized in Table S1. In addition, we also prepared surfactant-templated mesoporous silica MCM-41 and SBA-15, which have different mesostructured from KIE-6. Figure 1 shows TEM images of mesoporous silica materials as a catalytic supports. KIE-6-a, -b, -c, and -d have disordered and 3D-interconnected wormhole mesopore structure with different particle size of silica nanosphere framework. In contrast, SBA-15 and MCM-41 gave ordered 2D hexagonal mesostructure with cylindrical pores. Whereas SBA-15 has long cylindrical pores with length of about 1 micrometer, MCM-41 consists of oval-shaped secondary particles having short cylindrical pores with length of about 100 nm. Figures 2a,b present nitrogen sorption results of KIE-6-a, -b,-c, and -d. KIE-6-a, -b,-c, and -d have type IV isotherms with H2 hysteresis loops, which is commonly associated with ink-bottle pores or voids among close-packed spherical particles. As glycerol concentration or silica particle size increased, their hysteresis loop shifted toward higher P/Po (Fig. 2a), and pore diameter increased (Fig. 2b). Compared KIE-6-b with KIE-6-c, it was revealed that the increase in glycerol concentration of G-SN nanocomposites led to the increase in pore size and pore volume of KIE-6 with surface area maintained (Table S2). Moreover, compared KIE-6-a with KIE-6-d, it was confirmed that the increase in silica particle size resulted in the increase in pore size of KIE-6. However, surface area and pore volume decreased due to thicker pore wall of KIE-6-d than KIE-6-a (Table S2). Figures 2c,d show the nitrogen sorption results for SBA-15 and MCM-41. SBA-15 and MCM-41 have type IV isotherms with a H1 hysteresis loop, which is commonly associated with cylindrical pores. Whereas SBA-15 provided monomodal pore size distribution with pore diameter of 5.8 nm, MCM-41 had bimodal pore size distribution comprising small mesopores of 2.3 nm and large mesopores of about 64.0 nm. The large mesopores of MCM-41 originated from interstitial voids among oval-shaped secondary MCM-41 particles.

### NH_2_-functionalization of mesoporous silica supports

**Figure 3:**
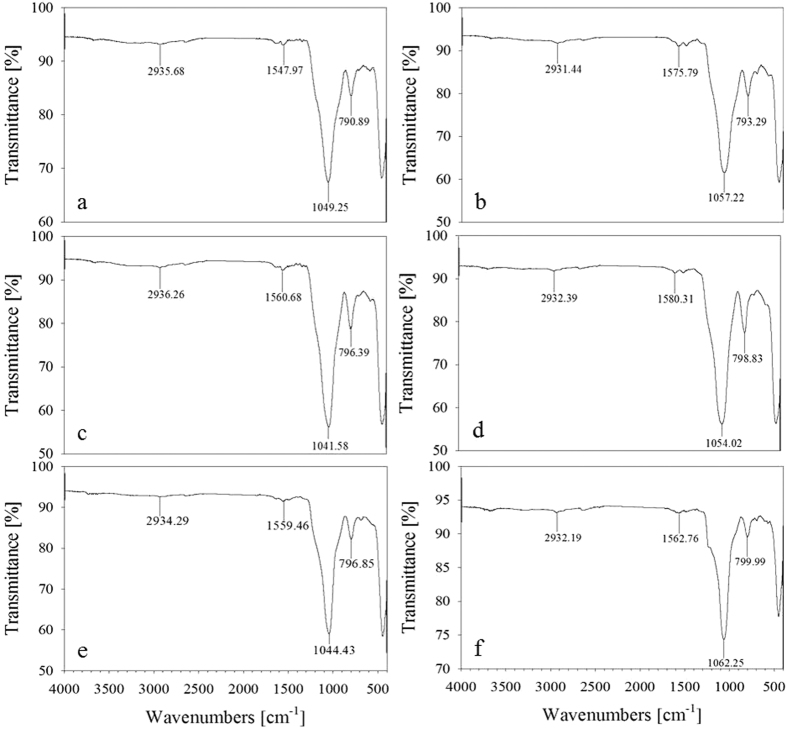
FT-IR spectra for NH_2_-functionalized mesoporous silica supports. (**a**) NH_2_-KIE-6-a, (**b**) NH_2_-KIE-6-b, (**c**) NH_2_-KIE-6-c, (**d**) NH_2_-KIE-6-d, (**e**) NH_2_-SBA-15, (**f**) NH_2_-MCM-41.

**Figure 4:**
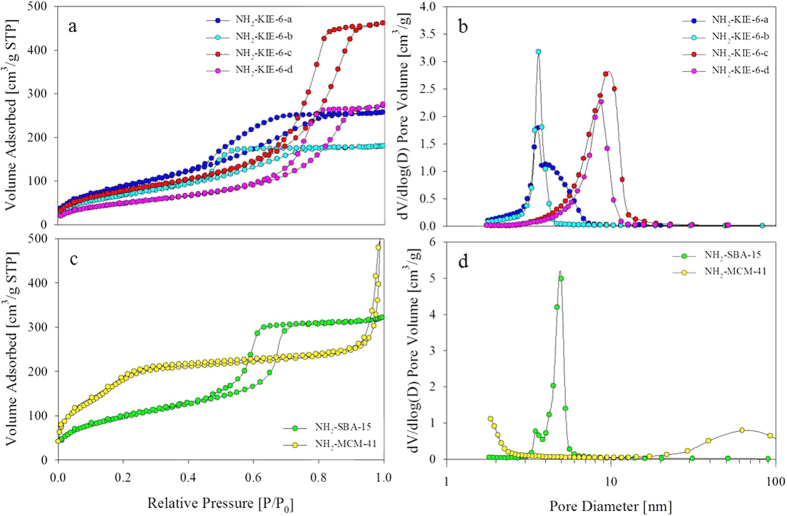
Nitrogen sorption results of NH_2_-KIE-6, NH_2_-SBA-15 and NH_2_-MCM-41. (**a,b**) NH_2_- KIE-6-a, -b, -c, -d. (**c,d**) NH_2_-SBA-15 and NH_2_-MCM-41. (**a,c**) Isotherms. (**b,d**) BJH desorption pore size distributions.

Before using mesoporous silica with various pore structures, the chemical modification of mesoporous silica surface was conducted to improve catalytic activity for formic acid decomposition. In general, amine or sodium formate is used as a basic additive to promote formic acid decomposition. However, considering separation issues of additives, we functionalized 3-aminopropyl groups on all of as-prepared mesoporous silica to increase surface basicity. Figure 3 shows FTIR spectra for NH_2_-functionalized KIE-6, SBA-15, and MCM-41. The peaks at 790–799 and 1041–1062 cm^−1^ for NH_2_-KIE-6, NH_2_-SBA-15 and NH_2_-MCM-41 correspond to symmetric and asymmetric stretching vibration of Si-O-Si. Furthermore, the bands at 1547 and 2935 cm^−1^ for NH_2_-KIE-6-a were attributed to N-H bending and C-H stretching vibration, 1575 and 2931 cm^−1^ for NH_2_-KIE-6-b, 1560 and 2936 cm^−1^ for NH_2_-KIE-6-c, 1580 and 2932 cm^−1^ for NH_2_-KIE-6-d, 1559 and 2934 cm^−1^ for NH_2_-SBA-15, and 1562 and 2932 cm^−1^ for NH_2_-MCM-41. On the basis of FTIR spectra, it was demonstrated that NH_2_ groups were successfully functionalized on all of mesoporous silica supports. Figure 4 shows nitrogen sorption isotherms and BJH desorption pore diameter distributions for NH_2_-KIE-6, NH_2_-SBA-15 and NH_2_-MCM-41 samples. After NH_2_ group functionalization, their BET surface area decreased from 846 m^2^/g to 326 m^2^/g for NH_2_-KIE-6-a, from 497 m^2^/g to 252 m^2^/g for NH_2_-KIE-6-b, from 452 m^2^/g to 289 m^2^/g for NH_2_-KIE-6-c, from 296 m^2^/g to 183 m^2^/g for NH_2_-KIE-6-d, from 730 m^2^/g to 363 m^2^/g for NH_2_-SBA-15, and from 1004 m^2^/g to 822 m^2^/g for NH_2_- MCM-41. Moreover, pore diameter of supports decreased from 4.8 nm to 3.6 nm for NH_2_-KIE-6-b, from 10.6 nm to 9.3 nm for NH_2_-KIE-6-c, from 9.5 nm to 8.7 nm for NH_2_-KIE-6-d, from 5.8 nm to 4.9 nm for NH_2_-SBA-15. The decrease in pore size and surface area after NH_2_-functionalization is another evidence for successful NH_2_-functionalization on the pore wall of mesoporous silica.

### Catalytic activity of Pd-MnO_x_/NH_2_-mesoporous-silica catalysts

To observe the effect of pore structure of mesoporous silica supports on catalytic activity for formic acid decomposition at room-temperature without additive, we prepared all of the catalysts under same condition (synthetic method, active metal, promoter, and content) except for the pore structure of silica supports. Pd(2 wt%)-MnO_x_(Mn basis 4 wt%)/NH_2_-mesoporous-silica catalysts were synthesized by reduction of palladium and manganese precursor solution, containing NH_2_-mesoporous silica supports, by NaBH_4_. Manganese oxide plays a significant role as a promoter for inhibiting of poisoning CO by dehydration reaction^27^,^33^. In addition, we conducted the inductively couples plasma atomic emission spectroscopy (ICP-AES) analyses for all the catalyst samples, and confirmed that the metal loading contents of the catalysts were almost consistent with the designed contents of metal loading (Table S4).

**Figure 5:**
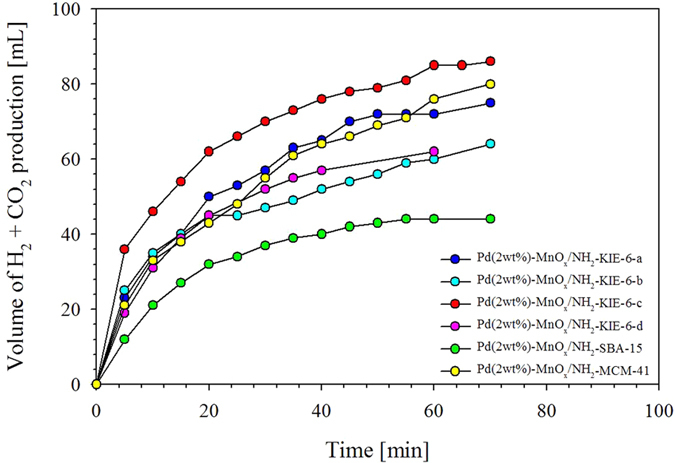
Formic acid decomposition activity of catalysts.

Figure 5 shows the catalytic performance of Pd and MnO_x_ nanoparticles supported on NH_2_ functionalized KIE-6, SBA-15 and MCM-41. The volume of production gas (H_2_ and CO_2_) and turnover frequency (TOF) at initial 10 min and 25 ^o^C for Pd(2 wt%)-MnO_x_(Mn basis 4 wt%)/NH_2_-KIE-6-a was 75 mL and 438.7 mol H_2_ mol catalyst^−1^ h^−1^, and Pd(2 wt%)-MnO_x_(Mn basis 4 wt%)/NH_2_-KIE-6-b produced 64 mL of H_2_ and CO_2_, and a TOF at initial 10 min and 25 ^o^C was 451.1 mol H_2_ mol catalyst^−1^ h^−1^. For Pd(2 wt%)-MnO_x_(Mn basis 4 wt%)/NH_2_-KIE-6-c catalyst, 86 mL of H_2_ and CO_2_ was produced. and TOF at initial 10 min was 593 mol H_2_ mol catalyst^−1^ h^−1^. In the case of Pd(2 wt%)-MnO_x_(Mn basis 4 wt%)/NH_2_-KIE-6-d, volume of production gas (H_2_ and CO_2_) and TOF at initial 10 min and 25 ^o^C was 62 mL and 400.0 mol H_2_ mol catalyst^−1^ h^−1^, respectively. Moreover, Pd(2 wt%)-MnO_x_(Mn basis 4 wt%)/NH_2_-SBA-15 produced 44 mL of H_2_ and CO_2_, and TOF at initial 10 min and 25 ^o^C was 271.0 mol H_2_ mol catalyst^−1^ h^−1^. For Pd(2 wt%)-MnO_x_(Mn basis 4 wt%)/ NH_2_-MCM-41 catalyst, 80 mL of H_2_ and CO_2_ was produced, and TOF at initial 10 min was 425.8 mol H_2_ mol catalyst^−1^ h^−1^. As a result, whereas Pd(2 wt%)-MnO_x_(Mn basis 4 wt%)/NH_2_-SBA-15 catalysts gave the lowest activity, Pd(2 wt%)-MnO_x_(Mn basis 4 wt%)/NH_2_-KIE-6-c catalysts provided the highest catalytic activity with excellent TOF of 593 mol H_2_ mol catalyst^−1^ h^−1^. It was demonstrated that the pore structure of supports significantly influenced the catalytic performance of formic acid decomposition. We deduce that 3D interconnected pore structure of mesoporous silica supports is more favorable to the mass transfer than 2D cylindrical mesopore structure, and the better mass transfer provides higher catalytic activity in formic acid decomposition. If the pore morphology of catalytic supports such as 3D wormhole or 2D cylinder is identical, large pore size combined with high pore volume is a crucial factor to achieve high catalytic performance. In addition, we investigated whether the selectivity and stability of the catalysts were also affected by the support pore structures. On the basis of the Fig. S2, hydrogen selectivity was calculated and estimated to be more than 99.9% for both Pd(2 wt%)-MnO_x_(Mn basis 4 wt%)/KIE-6-c and Pd(2 wt%)-MnO_x_(Mn basis 4 wt%)/MCM-41. Figure S3 presents the stability results for Pd(2 wt%)-MnO_x_(Mn basis 4  wt%)/KIE-6-c and Pd(2 wt%)-MnO_x_(Mn basis 4 wt%)/MCM-41, indicating that both of the catalysts provided good recyclability. Based on such results, it was confirmed that the catalytic activity (TOF) is significantly influenced by the pore properties of catalyst supports, however the catalysts provided good selectivity and recyclability regardless of support pore structure. However, to clearly verify the effect of support pore structure on catalytic activity (TOF), it is necessary to investigate the effect of Pd and MnOx nanoparticle size and electronic state on catalytic performance.

### Characterization of Pd-MnO_x_/NH_2_-SiO_2_ catalysts

**Figure 6:**
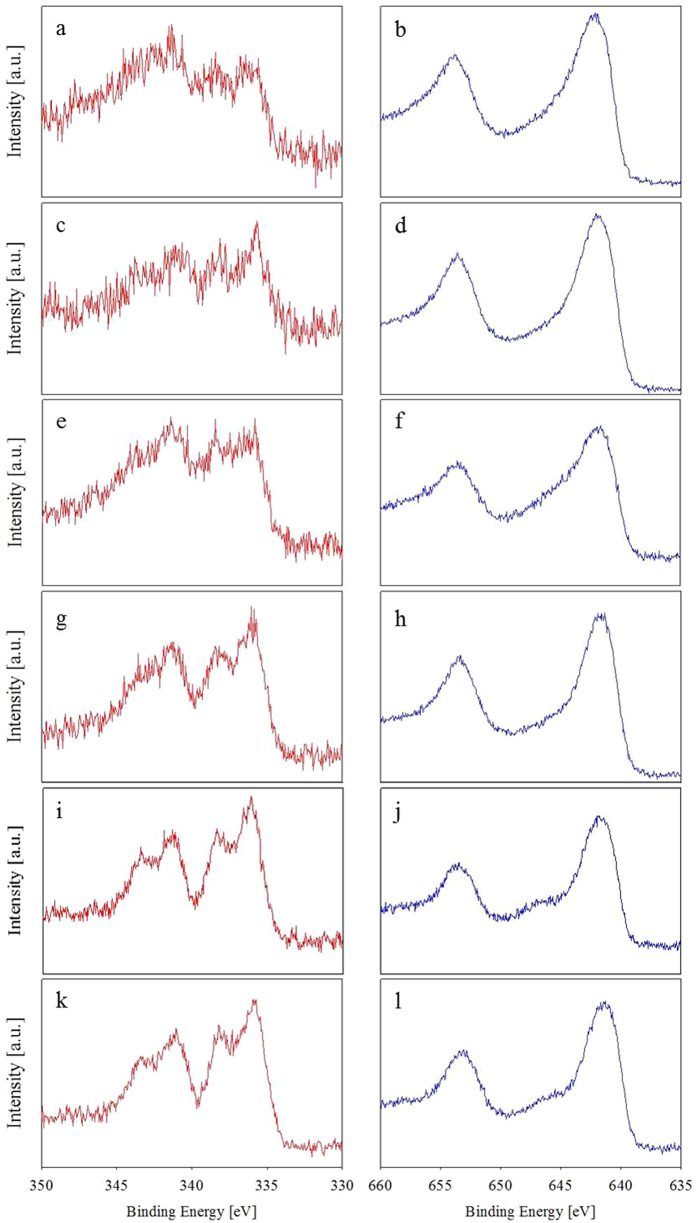
XPS spectra of catalysts. (**a,b**) Pd(2wt%)-MnO_x_(Mn basis 4 wt%)/ KIE-6-a, (**c,d**) Pd(2wt%)-MnO_x_(Mn basis 4 wt%)/ KIE-6-b, (**e,f**) Pd(2wt%)-MnO_x_(Mn basis 4 wt%)/ KIE -6-c, (**g,h**) Pd(2wt%)-MnO_x_(Mn basis 4 wt%)/ KIE-6-d, (**i,j**) Pd(2wt%)-MnO_x_(Mn basis 4 wt%)/SBA-15, (**k,l**) Pd(2wt%)-MnO_x_(Mn basis 4 wt%)/MCM-41. (**a,c,e,g,i,k**) Pd 3d spectra. (**b,d,f,h,j,l**) Mn 2p spectra.

**Figure 7:**
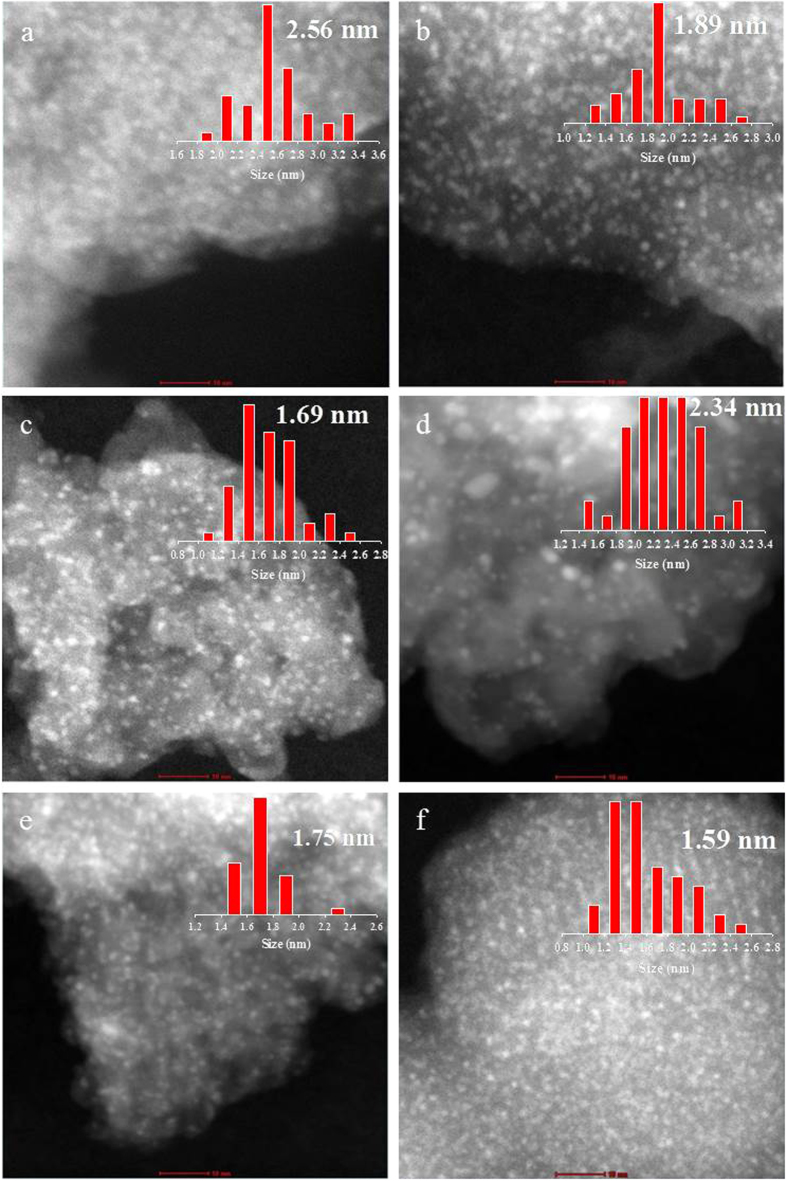
STEM images of catalysts. (**a**) Pd(2wt%)-MnO_x_(Mn basis 4 wt%)/NH_2_-KIE-6-a, (**b**) Pd(2wt%)-MnO_x_(Mn basis 4 wt%)/NH_2_-KIE-6-b, (**c**) Pd(2wt%)-MnO_x_(Mn basis 4 wt%)/NH_2_-KIE-6-c, (**d**) Pd(2wt%)-MnO_x_(Mn basis 4 wt%)/ NH_2_-KIE-6-d, (**e**) Pd(2wt%)-MnO_x_(Mn basis 4 wt%)/NH_2_-SBA-15, (**f**) Pd(2wt%)-MnO_x_(Mn basis 4 wt%)/ NH_2_-MCM-41.

To verify the electronic state and particle size of Pd and MnOx in Pd(2wt%)-MnO_x_(Mn basis 4 wt%)/NH_2_-SiO_2_ catalysts, we carried out X-ray photoelectron spectroscopy (XPS) and high-angle annular dark-field scanning transmission electron microscopy (HAADF-STEM) analyses. Figure 6 shows XPS (Pd 3d and Mn 2p) spectra for Pd(2 wt%)-MnO_x_(Mn basis 4 wt%)/NH_2_-SiO_2_ catalysts. As shown in Fig. 6a, intense peaks for Pd(2 wt%)-MnO_x_(Mn basis 4 wt%)/NH_2_-KIE-6-a are observed at 336.7 eV, 338.4 eV and 341.3 eV, which are ascribed to Pd(0) 3d_5/2_ state, Pd(0) 3d_3/2_ state and Pd(II) 3d_5/2_ state, respectively. Figure 6b exhibits Mn 2p spectra for Pd(2 wt%)-MnO_x_(Mn basis 4 wt%)/NH_2_-KIE-6-a. Broad peaks centered at 642.1 eV are ascribed to Mn 2p_3/2_, consisting of Mn^2+^, Mn^3+^ and Mn^4+^, indicating that the chemical state of Mn is MnOx. In addition, the XPS spectra for Pd-MnO_x_/NH_2_-KIE-6-b, Pd-MnO_x_/NH_2_-KIE-6-c, Pd-MnO_x_ /NH_2_-KIE-6-d, Pd-MnO_x_/NH_2_-SBA-15 and Pd-MnO_x_/ NH_2_-MCM-41 are given in Fig. 6c–l. All of the Pd-MnOx/NH_2_-mesoporous-SiO_2_ provided almost same pattern of Pd 3d and Mn 2p XPS spectra. Thus it was demonstrated that pore structure of mesoporous silica supports did not influence the electronic state of Pd and Mn in the catalysts (Figs 6 and S5). As shown in Fig. 7, the particle size distribution of Pd and MnO_x_ nanoparticles in Pd(2 wt%)-MnO_x_(Mn basis 4 wt%)/NH_2_-mesoporous-SiO_2_ catalysts were analyzed by HAADF-STEM. Pd-MnO_x_/NH_2_-KIE-6-a, Pd-MnO_x_/NH_2_-KIE-6-b, Pd-MnO_x_/NH_2_-KIE-6-c, Pd-MnO_x_/NH_2_-KIE-6-d, Pd-MnO_x_/NH_2_-SBA-15 and Pd-MnO_x_/NH_2_-MCM-41 have average particle size of 2.56 nm (Fig. 7a), 2.89 nm (Fig. 7b), 1.69 nm (Fig. 7c), 2.34 nm (Fig. 7d), 1.75 nm (Fig. 7e) and 1.59 nm (Fig. 7f), respectively. The remarkable difference in nanoparticle size was not observed. XRD analyses were also conducted to calculate the nanoparticle size for all of the catalysts. However, XRD patterns for all the samples gave only a broad peak associated with amorphous silica supports (Fig. S4), and the peaks for Pd, PdO or MnOx phase were not detected. As shown in TEM images (Fig. 7), Pd and MnOx nanoparticles are too small to be detected by XRD analyses. Meanwhile, comparing Pd-MnO_x_/NH_2_-SBA-15 with Pd-MnO_x_/NH_2_-KIE-6-a, Pd-MnO_x_/NH_2_-KIE-6-b, and Pd-MnO_x_/NH_2_-KIE-6-d, although nanoparticle size for Pd-MnO_x_/NH_2_-SBA-15 is smaller than that for Pd-MnO_x_/NH_2_-KIE-6-a, Pd-MnO_x_/NH_2_-KIE-6-b, and Pd-MnO_x_/NH_2_-KIE-6-d, the catalytic performance of Pd-MnO_x_/NH_2_-SBA-15 is much lower, which indicates that there is no correlation between nanoparticle size (Fig. 7) and catalytic activity (Fig. 5). On the basis of Figs 5, 6, 7, it was concluded that the electronic state and nanoparticle size of Pd and MnOx in Pd(2 wt%)-MnO_x_(Mn basis 4 wt%)/NH_2_-SiO_2_ were not influential factors for the variation of formic acid decomposition performance.

### Correlation between catalytic activity and support pore properties

**Figure 8:**
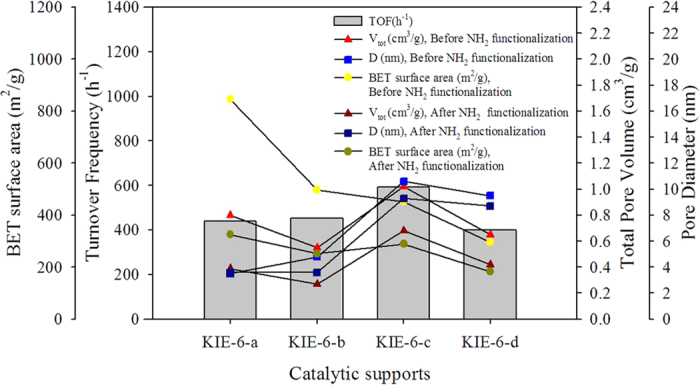
Correlation between TOF and pore properties of catalytic supports. (TOF values are for Pd(2wt%)-MnO_x_ (Mn basis 4 wt%)/NH_2_-KIE-6 prepared with each KIE-6 support.)

To demonstrate effect of support pore properties on catalytic activity, we investigated the correlation between TOF and support pore properties such as total pore volume, pore diameter and BET surface area (Fig. 8). Compared with KIE-6-a, KIE-6-b and KIE-6-d, KIE-6-c exhibits much higher catalytic activity for additive-free formic acid decomposition at room temperature. In addition, KIE-6-c has higher total pore volume and larger pore diameter than the other catalyst supports. After NH_2_ group functionalization, their total pore volume decreased from 0.8 cm^3^/g to 0.39 cm^3^/g for NH_2_-KIE-6-a, from 0.55 cm^3^/g to 0.27 cm^3^/g for NH_2_-KIE-6-b, from 1.02 cm^3^/g to 0.68 cm^3^/g for NH_2_-KIE-6-c, and from 0.65 cm^3^/g to 0.42 cm^3^/g for NH_2_-KIE-6-d, and pore diameter of supports also decreased. As shown in Fig. 8, even after NH_2_ functionalization, KIE-6-c provided much higher pore volume and larger pore size than the other catalyst supports. However, although KIE-6-c provided the highest TOF in formic acid decomposition, its BET surface area was much lower than KIE-6-a and KIE-6-b, which demonstrating that the effect of pore volume and size on TOF is much more dominant than the effect of surface area. In general, the surface area is a very important factor for catalytic activity. However, in case of formic acid decomposition at room temperature (liquid phase reactants), even if the surface area of support is very high, lower TOF can be obtained due to low pore volume and small pore size. That is, the mass transfer for the access of liquid phase formic acid to active sites and the release of products from active sites is considered to be a very important factor in formic acid decomposition at room temperature.

Comparing KIE-6-c with KIE-6-d, although KIE-6-c and KIE-6-d have similar pore size, the pore volume of KIE-6-c is much higher than KIE-6-d, which leads to much higher TOF of KIE-6-c. Comparing KIE-6-a with KIE-6-d, KIE-6-d has much larger pore size than KIE-6-a, but they shows a little difference in total pore volume, which means that the number of pores for KIE-6-a is larger or pore wall of KIE-6-d is thicker. As a result, the TOF of KIE-6-a was similar to that of KIE-6-d. Consequently, it was revealed that large pore size combined with high pore volume played an important role in the excellent catalytic activity of KIE-6-c supports.

Moreover, comparing KIE-6-a with SBA-15, we demonstrated that 3D interconnected pore morphology is more favorable to formic acid decomposition in comparison with 2D ordered pore morphology. On the basis of Table S2 and Fig. 8, although SBA-15 has larger pore size and higher pore volume than KIE-6-a, SBA-15 showed much lower TOF than KIE-6-a. This is because KIE-6-a has 3D interconnected pore structure, whereas SBA-15 has very long 2D cylindrical pores with length of about 1 micrometer (Fig. 1) which highly suppresses the mass transfer of reactants and products. Comparing the morphological pore structure of MCM-41 with SBA-15 (Fig. 1), whereas SBA-15 has long cylindrical pores, MCM-41 has short cylindrical pores with length of about 100 nm and interstitial macropores among MCM-41 particles. Because of such a pore morphology of MCM-41 being favorable to the mass transfer, MCM-41 gave much higher TOF than SBA-15 in spite of larger primary pore size of SBA-15. That is, even though they have the same 2D cylindrical pore structure, the length of cylindrical pores or the presence of macropores significantly influences their catalytic activity. In summary, if the morphological pore structures of catalytic supports are identical, large pore size combined with high pore volume is a crucial factor to achieve high catalytic performance in formic acid decomposition. However, if the catalytic supports have the pore morphology being more favorable to the mass transfer, they could provide higher catalytic activity although they have lower pore volume and smaller pore size.

Furthermore, we investigated the effect of length of NH_2_ functional groups on the catalytic activity by conducting the functionalization of –(CH_2_)_3_NHCH_2_CH_2_NH_2_ groups on KIE-6-c supports (diamine-KIE-6-c). Whereas the NH_2_-KIE-6-c supports have –(CH_2_)_3_NH_2_ groups on KIE-6-c surface, diamine-KIE-6-c supports have longer –(CH_2_)_3_NHCH_2_CH_2_NH_2_ groups on KIE-6-c surface but have more amine groups. Figure S6 shows BET surface area, pore diameter and total pore volume for NH_2_-6-c and diamine-6-c, and TOF for Pd(2 wt%)-MnO_x_(Mn basis 4 wt%)/NH_2_-6-c and Pd(2 wt%)-MnO_x_(Mn basis 4 wt%)/diamine-6-c. Substituting –(CH_2_)_3_NH_2_ functional groups (NH_2_-KIE-6-c) with –(CH_2_)_3_NHCH_2_CH_2_NH_2_ functional groups (diamine-KIE-6-c), BET surface area of the catalytic support decreased from 289 m^2^/g and 204 m^2^/g, and pore diameter decreased from 9.3 nm and 7.7 nm, and total pore volume decreased from 0.68 cm^3^/g and 0.55 cm^3^/g. In addition, Pd(2 wt%)-MnO_x_(Mn basis 4 wt%)/diamine-KIE-6-c produced 39 mL of H_2_ and CO_2_, and a TOF at initial 10 min and 25 ^o^C was 167.7 mol H_2_ mol catalyst^−1^ h^−1^, which is much lower than Pd(2 wt%)-MnO_x_(Mn basis 4 wt%)/NH_2_-6-c catalysts. In spite of more amine groups as a basic site, Pd(2 wt%)-MnO_x_(Mn basis 4 wt%)/diamine-KIE-6-c gave much lower catalytic activity. The lower catalytic activity of Pd(2 wt%)-MnO_x_(Mn basis 4 wt%)/diamine-KIE-6-c is attributed to lower pore volume, smaller pore size and surface area. In addition, longer –(CH_2_)_3_NHCH_2_CH_2_NH_2_ groups on KIE-6-c surface considerably hinders the access of formic acid to active sites.

## Discussion

To investigate correlation between support pore structure and catalytic activity in formic acid decomposition, we used mesoporous silica KIE-6, MCM-41, and SBA-15 as a catalytic support. Compared to MCM-41 and SBA-15, KIE-6 is more suitable support materials to investigate the effect of support pore structure, because pore size of KIE-6 is easily controlled by glycerol concentration in glycerol-silica nanosphere (G-SN) nanocomposites.

Using the mesoporous silica materials, we prepared Pd(2wt%)-MnO_x_(Mn basis 4 wt%)/NH_2_-KIE-6-a, Pd-MnO_x_/NH_2_-KIE-6-b, Pd-MnO_x_/NH_2_-KIE-6-c, Pd-MnO_x_/NH_2_-KIE-6-d, Pd-MnO_x_/NH_2_-MCM-41, Pd-MnO_x_/NH_2_-SBA-15, and estimated their catalytic activity for formic acid decomposition at room temperature. As a result, whereas Pd(2 wt%)-MnO_x_(Mn basis 4 wt%)/NH_2_-SBA-15 catalysts gave the lowest activity, Pd(2 wt%)-MnO_x_(Mn basis 4 wt%)/NH_2_-KIE-6-c catalysts provided the highest catalytic activity with excellent TOF of 593 mol H_2_ mol catalyst^−1^ h^−1^.

The remarkable difference in the electronic state and nanoparticle size of Pd and MnO_x_ in Pd(2wt%)-MnO_x_(Mn basis 4 wt%)/NH_2_-SiO_2_ was not observed. Thus it was concluded that the electronic state and nanoparticle size of Pd and MnOx were not influential factors for the variation of formic acid decomposition performance, and the different catalytic activity for the Pd(2 wt%)-MnO_x_(Mn basis 4 wt%)/NH_2_-SiO_2_ catalysts originated from different pore structure of supports. In summary, if the morphological pore structures of catalytic supports are identical, large pore size combined with high pore volume is a crucial factor to achieve high catalytic performance in formic acid decomposition. However, if the catalytic supports have the pore morphology being more favorable to the mass transfer, they could provide higher catalytic activity although they have lower pore volume and smaller pore size.

## Methods

### Synthesis of silica sol

Polymeric silica sol was synthesized by acid catalyzed hydrolysis and condensation of tetraethylorthosilicate (TEOS: 98%, Aldrich). A mixture of TEOS, water and nitric acid was vigorously stirred at room temperature. The molar ratio of TEOS:H_2_O:HNO_3_ was at 1:5.8:0.08. Then additional deionized H_2_O was carefully added into the mixture under vigorous stirring to adjust the final solution volume to 500 mL. The final mixture was refluxed for 8 h at 80 ^o^C. As a result, polymeric silica sol was synthesized with particle diameter under 2 nm.

Colloidal silica sol was prepared under base-catalyzed condition at a TEOS:NH_3_:H_2_O:EtOH molar ratio of 1:0.084:53.6:40.7. A mixture of NH_3_ and H_2_O was carefully added to a mixture of TEOS and EtOH under vigorously stirring at 50 ^o^C. The final mixture solution was refluxed for 3 h at 50 ^o^C. As a result, colloidal silica sol with particle diameter of about 5 nm was synthesized. In addition, colloidal silica sol with particle diameter of about 10 nm was prepared by the same method as 5 nm silica sol except for a reactant molar ratio and reflux time. The molar ratio of TEOS:NH_3_:H_2_O:EtOH for the silica sol with particle diameter of about 10 nm was 1:0.0084:19.8:2.2, and the mixture solution was refluxed for 3 days at at 50 ^o^C.

### Synthesis of mesoporous silica KIE-6

To investigate the effect of support pore size on formic acid decomposition performance without additives at room temperature, we prepared glycerol-templated mesoporous silica KIE-6 with different pore size. In a typical synthesis, the nanocomposite sol was prepared by mixing polymeric silica sol or colloidal silica sol with glycerol and sulfuric acid, and then the nanocomposite sol was dried at 150 ^o^C for 24 h in air for solvent elimination and precarbonization of glycerol. The nanocomposite of silica nanoparticles and precarbonized glycerol was calcined at 550 ^o^C for 2 h in air, resulting in mesoporous silica KIE-6. The KIE-6-a, KIE-6-b, KIE-6-c, and KIE-6-d were prepared by changing the composition of the nanocomposite sol. In case of KIE-6-a, the nanocomposite sol was prepared by adding pure glycerol (99%, DUKSAN) 9.3 g and sulfuric acid (10 wt% of glycerol) into 100 mL of polymeric silica sol, and the nanocomposite sol for mesoporous silica KIE-6-c was prepared by mixing 4 g of pure glycerol, sulfuric acid (10 wt% of glycerol) and 30 mL of colloidal silica sol with particle size of 5 nm. The nanocomposite sol for KIE-6-d was synthesized by adding 33.3 g of pure glycerol and sulfuric acid (10 wt% of glycerol) into 100 mL of colloidal silica sol with particle size of 10 nm. In addition, mesoporous silica KIE-6-b was prepared by drying and calcination of colloidal silica sol with particle size of 5 nm without the addition of pure glycerol and sulfuric acid.

### Synthesis of amine-functionalized KIE-6

The amine-functionalization of the mesoporous silica KIE-6 was carried out with 3-aminopropyl trimethoxysilane (APTMS: Aldrich, 97%). 0.4 g of KIE-6 was added into 100 mL toluene, followed by addition of 1 mL of APTMS. The mixture was refluxed without stirring for 3 h at 110 ^o^C, and then the product was filtered and washed repeatedly with toluene for removal of unreacted APTMS. The NH_2_-functionalized KIE-6 (NH_2_-KIE-6) was prepared by drying for 24 h at room-temperature.

### Synthesis of Pd-MnO_x_/NH_2_-KIE-6 catalysts for formic acid decomposition

For preparation of Pd(2 wt%)MnO_x_(Mn basis 4 wt%) catalysts supported on NH_2_-KIE-6-a, -b, -c, and -d, 0.0288 g of manganese(II) chloride tetrahydrate (Aldrich) and 0.04 g of 10 wt% palladium(II) nitrate aqueous solution (PM RESEARCH) were added to 10 mL of deionized H_2_O, followed by the addition of 0.18 g of NH_2_-KIE-6-a, -b, -c, or -d supports. Afterward, 2 mL of 0.85 M NaBH_4_ aqueous solution was added into the mixture with vigorous stirring, and was stirred for 1 h at room temperature. After centrifugation, the obtained Pd(2 wt%)MnO_x_(Mn basis 4 wt%)/NH_2_-KIE-6-a, -b, -c, and -d were washed with deionized H_2_O, and dried at room temperature.

### Synthesis of diamine-functionalized KIE-6-c

The diamine functionalization of the mesoporous silica KIE-6 was carried out with N-(2-aminoethyl)-3-(trimethoxysilyl) propylamine (AETMSPA, Aldrich). 0.4 g of KIE-6 was added into 100 mL toluene, followed by addition of 1.3 mL of AETMSPA. The mixture was refluxed without stirring for 3 h at 110 ^o^C, and then the product was filtered and washed repeatedly with toluene. The diamine-functionalized KIE-6 (di-NH_2_-KIE-6-c) were prepared by drying for 24 h at room-temperature.

### Synthesis of Pd-MnOx/di-NH_2_-KIE-6 catalyst for formic acid decomposition

To investigate the effect of surface basicity of supports on formic acid decomposition performance, Pd(2 wt%)MnO_x_(Mn basis 4 wt%) catalysts supported on di-NH_2_-KIE-6-c was also prepared. The synthesis procedure was the same as that for Pd(2 wt%)MnO_x_(Mn basis 4 wt%)/NH_2_-KIE-6 except for using di-NH_2_-KIE-6-c as a catalyst support.

### Decomposition of formic acid at room temperature

The hydrogen production from formic acid (FA) solution was carried out in a 100 mL Teflon-lined stainless steel reactor at room temperature (25 ^o^C). A gas burette system filled with water was connected to the outlet of the reactor to measure the volume of produced gas. 0.055 g of catalyst was located in the Teflon-lined reactor, followed by a nitrogen pure for 30 min. Afterward, the reaction started when a mixture of 10 mL of deionized H_2_O and 0.19 mL of formic acid (95%, Aldrich) was injected into the reactor through a rubber septum.

### Characterization

The pore propertied of KIE-6 and NH_2_-KIE-6 were taken by nitrogen sorption tests with a Micromeritics ASAP 2420 instrument. Degassing of samples was carried out at 200 ^o^C for 5 h. For characterization of the Pd(2 wt%)-MnO_x_/NH_2_-KIE-6, Pd(2 wt%)-MnO_x_/NH_2_-MCM-41, and Pd(2 wt%)-MnO_x_/NH_2_-SBA-15 catalysts, we carried out X-ray photoelectron spectroscopy (XPS), x-ray diffraction (XRD), inductively couples plasma atomic emission spectroscopy (ICP-AES), fourier transform infrared (FTIR), transmission electron microscopy (TEM), and scanning transmission electron microscopy (STEM) analyses. TEM and STEM analyses were conducted by using a FEI/TECNAI G2 instrument. XPS and FTIR measurements were performed using a Kratos 165XP spectrometer and a Thermo Nicolet 5700 instrument. The composition of produced gas was analyzed using a gas chromatography (GC, Agilent 6890) equipped with a thermal conductivity detector(TCD) and carboxen 1010 PLOT fused silica capillary column (30 m × 0.53 mm, SUPELCO).

## Additional Information

**How to cite this article**: Jin, M.-H. *et al*. Mesoporous Silica Supported Pd-MnO_x_ Catalysts with Excellent Catalytic Activity in Room-Temperature Formic Acid Decomposition. *Sci. Rep.* **6**, 33502; doi: 10.1038/srep33502 (2016).

## Supplementary Material

Supplementary Information
