# Supplementary material for: Mesoporous Silica Supported Pd-MnOx Catalysts with Excellent Catalytic Activity in Room-Temperature Formic Acid Decomposition
Source: Sci Rep. 2016 Sep 26;6:33502. doi: 10.1038/srep33502 (PMC5036166; doi:10.1038/srep33502)
Supplement: Supplementary Information [file srep33502-s1.doc]

**Supplementary Information**

**Mesoporous Silica Supported Pd-MnO_x_ Catalysts with Excellent Catalytic Activity in Room-Temperature Formic Acid Decomposition**

**Min-Ho Jin ^1, 2^, Duckkyu Oh ^1^, Ju-Hyoung Park^3^, Chun-Boo Lee ^1^, Sung-Wook Lee^1^, Jong-Soo Park ^1^, Kwan-Young Lee^2,*^, Dong-Wook Lee^1,*^**

**^1^** Advanced Materials and Devices Laboratory, Korea Institute of Energy Research (KIER),

*152 Gajeongro, Yuseong, Daejeon 305-343, Republic of Korea,*

***^2^****Department of Chemical and Biological Engineering, Korea University,*

*Sungbuk-gu, Seoul 136-701, Republic of Korea*

**^3^** Clean Fuel Laboratory, Korea Institute of Energy Research (KIER),

*152 Gajeongro, Yuseong, Daejeon 305-343, Republic of Korea*

** corresponding author:* [*dwlee99@kier.re.kr*](mailto:dwlee99@kier.re.kr)*,* [*kylee@korea.ac.kr*](mailto:kylee@korea.ac.kr)

**SUPPORTING TABLES**

Table S1. Synthesis conditions of KIE-6 samples.

| Sample name | Silica  framework | Glycerol  type | Glycerol/silica weigh ratio | Template  elimination method |
| --- | --- | --- | --- | --- |
| KIE-6-a | polymeric silica | pure | 17.7 | calcination |
| KIE-6-b | 5nm silica | Pure | 0 | calcination |
| KIE-6-c | 5nm silica | Pure | 38.4 | calcination |
| KIE-6-d | 10nm silica | pure | 19.1 | calcination |

Table S2. Pore properties for KIE-6, SBA-15, MCM-41, NH_2_-KIE-6, NH_2_-SBA-15 and NH_2_-MCM-41 obtained by nitrogen sorption tests.

| Sample name | SA_BET_  [m^2^/g]^a^ | SA_micro_  [m^2^/g]^b^ | V_tot_  [cm^3^/g]^c^ | D_a_  [nm]^d^ | D_p_  [nm]^e^ |  |
| --- | --- | --- | --- | --- | --- | --- |
| KIE-6-a | 846 | 0 | 0.80 | 3.6 | 3.5 and 4.8 |  |
| KIE-6-b | 497 | 23 | 0.55 | 4.1 | 4.8 |  |
| KIE-6-c | 452 | 32 | 1.02 | 8.5 | 10.6 |  |
| KIE-6-d | 296 | 28 | 0.65 | 8.5 | 9.5 |  |
| SBA-15 | 730 | 199 | 0.92 | 5.3 | 5.8 |  |
| MCM-41 | 1004 | 841 | 1.28 | 4.5 | 2.3 and 64.0 |  |
|  |  |  |  |  |  |  |
| NH_2_-KIE-6-a | 326 | 0 | 0.39 | 3.9 | 3.6 and 4.0 |  |
| NH_2_-KIE-6-b | 252 | 0 | 0.27 | 3.5 | 3.6 |  |
| NH_2_-KIE-6-c | 289 | 0 | 0.68 | 8.0 | 9.3 |  |
| NH_2_-KIE-6-d | 183 | 0 | 0.42 | 7.5 | 8.7 |  |
| NH_2_-SBA-15 | 363 | 29 | 0.49 | 4.7 | 4.9 |  |
| NH_2_-MCM-41 | 822 | 732 | 0.77 | 9.4 | 1.8 and 62.6 |  |

^a^ BET surface area

^b^ micropore surface area calculated from a t-plot

^c^ total pore volume taken from the volume of nitrogen adsorbed at P/Po=0.995

^d^ BJH desorption average pore diameter

^e^ BJH peak pore diameter

Table S3. Catalytic activity comparison of various heterogeneous catalysts for room-temperature formic acid decomposition without additives.

| Catalysts | Formic acid concentration  [mmol] | additives | Reaction temperature [^o^C] | TOF  at 10 min ^a^  [h^-1^] | Reference ^b^ |  |
| --- | --- | --- | --- | --- | --- | --- |
| Pd(2wt%)-MnO_x_  /NH_2_-KIE-6-c | 5 | - | 25 | 593.6 | This work |  |
| Pd(2wt%)-MnO_x_  /NH_2_-SBA-15 | 5 |  | 25 | 271.0 | This work |  |
| Pd(2wt%)-MnO_x_  /NH_2_-MCM-41 | 5 |  | 25 | 425.8 | This work |  |
| Ag@Pd(10wt%)/  Vulcan carbon | 10 | - | 20 | 26.0 | [13] |  |
| Pd(3.4wt%)/  NH_2_-SBA-15 | 10 | - | 25 | 293.0 | [14] |  |
| Au(4.6wt%)Pd(2.8wt%-MnO_x_/ZIF-8-rGO | 5 | - | 25 | 382.1 | [27] |  |
| Pd(38.7wt%)  Ag(36.3wt%)/MIL-100 | 10 | - | 25 | 20.5 | [29] |  |
| Pd(8wt%)/CN | 10 | - | 15 | 71.0 | [30] |  |
| Pd(1.82wt%)-MnO_x_  /NH_2_-silica gel | 2.65 | - | 20 | 248.7 | [31] |  |
| Pd(4.6wt%)  Ag(9.3wt%)/TiO_2_ | 5 | - | 27 | 190.1 | [32] |  |
| Pd/PDA-rGO | 6 | - | 25 | 216.0^c^ | [35] |  |
| Co(3wt%)Au(3.5wt%)Pd(3.5wt%)/Vulcan XC-72 | 5 | - | 25 | 80.0 | [36] |  |

^a^ TOF was calculated at initial 10 min of reaction time by using this equation (TOF=mole of produced hydrogen gas per hour/mole of metal catalyst).

^b^ see the references in the main text.

^c^ TOF was calculated at initial 18.5 min of reaction time by using this equation (TOF=mole of produced hydrogen gas per hour/mole of metal catalyst).

Table S4. The mass fraction of Pd and Mn in catalysts measured by inductively coupled plasma atomic emission spectroscopy (ICP-AES).

| Catalysts | Molar percent of Pd [%] | Molar percent of Mn [%] |
| --- | --- | --- |
| Pd(2wt%)-MnO_x_  /NH_2_-KIE-6-a | 2.03 | 3.92 |
| Pd(2wt%)-MnO_x_  /NH_2_-KIE-6-b | 1.97 | 3.83 |
| Pd(2wt%)-MnO_x_  /NH_2_-KIE-6-c | 1.92 | 3.87 |
| Pd(2wt%)-MnO_x_  /NH_2_-KIE-6-d | 2.07 | 3.71 |
| Pd(2wt%)-MnO_x_  /NH_2_-SBA-15 | 1.93 | 3.94 |
| Pd(2wt%)-MnO_x_  /NH_2_-MCM-41 | 1.94 | 4.13 |

**SUPPORTING FIGURES**


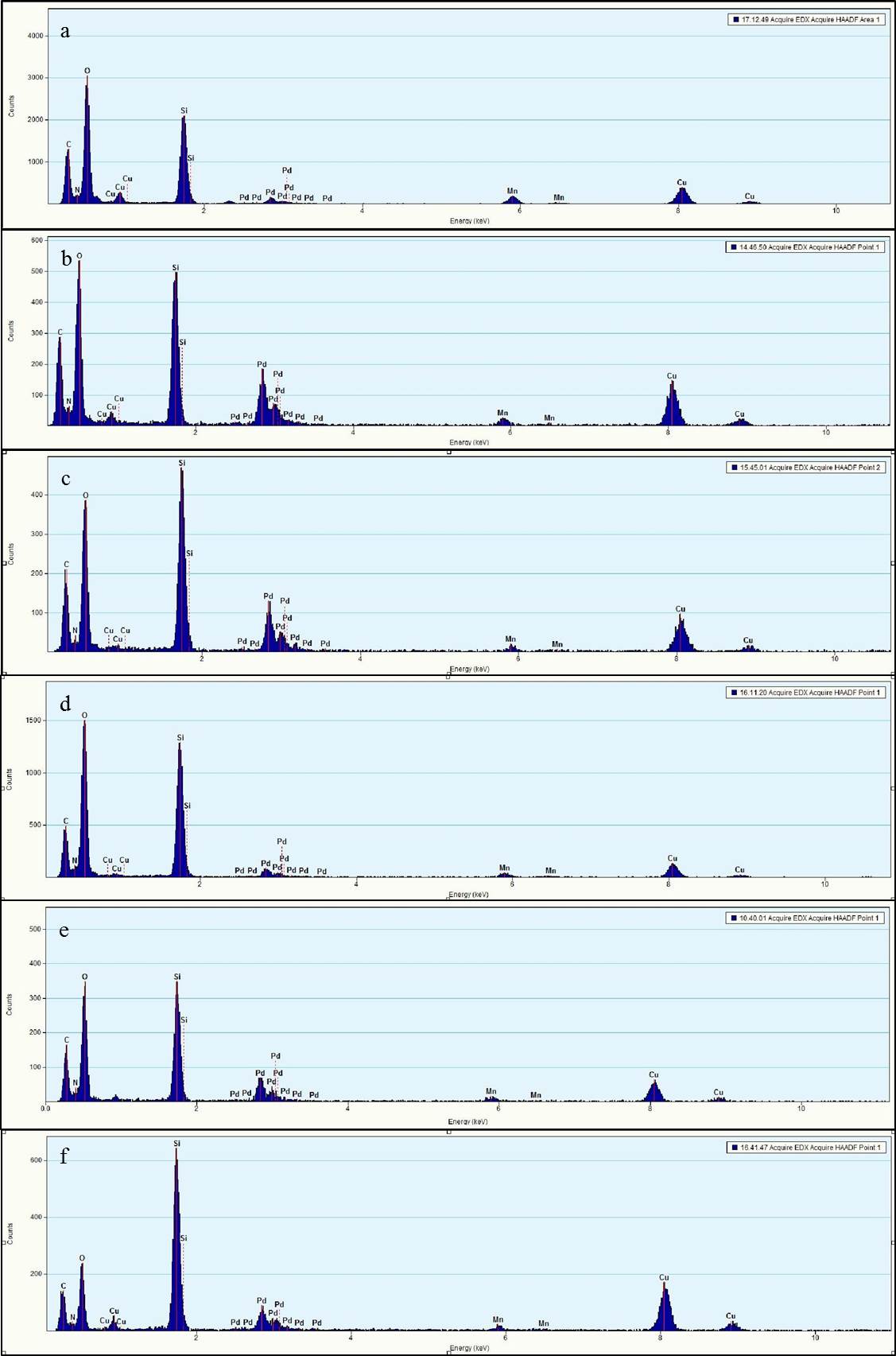


**Figure S1. EDS spectra of catalysts.** (a) Pd(2wt%)-MnO_x_(Mn basis 4 wt%)/NH_2_-KIE-6-a, (b) Pd(2wt%)-MnO_x_(Mn basis 4 wt%)/NH_2_-KIE-6-b, (c) Pd(2wt%)-MnO_x_(Mn basis 4 wt%)/NH_2_-KIE-6-c, (d) Pd(2wt%)-MnO_x_(Mn basis 4 wt%)/ NH_2_-KIE-6-d, (e) Pd(2wt%)-MnO_x_(Mn basis 4 wt%)/NH_2_-SBA-15, (f) Pd(2wt%)-MnO_x_(Mn basis 4 wt%)/ NH_2_-MCM-41.


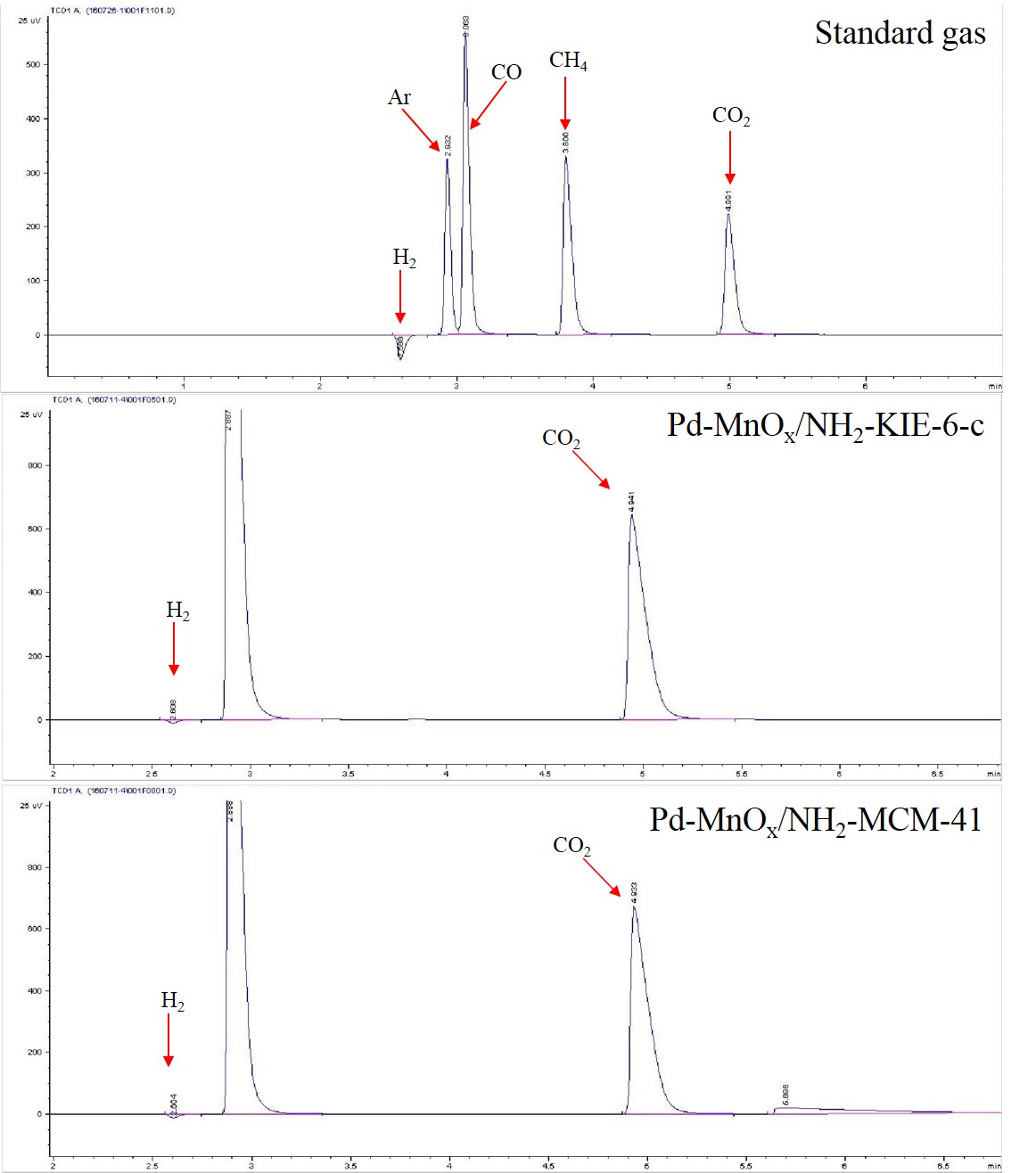


**Figure S2. Gas chromatography data of standard gas and produced gas.** The gases was analyzed by gas chromatography (Agilent 6890) with a carboxen 1010 PLOT fused silica capillary column (30 m × 0.53 mm, SUPELCO) and thermal conductivity detector(TCD).

**
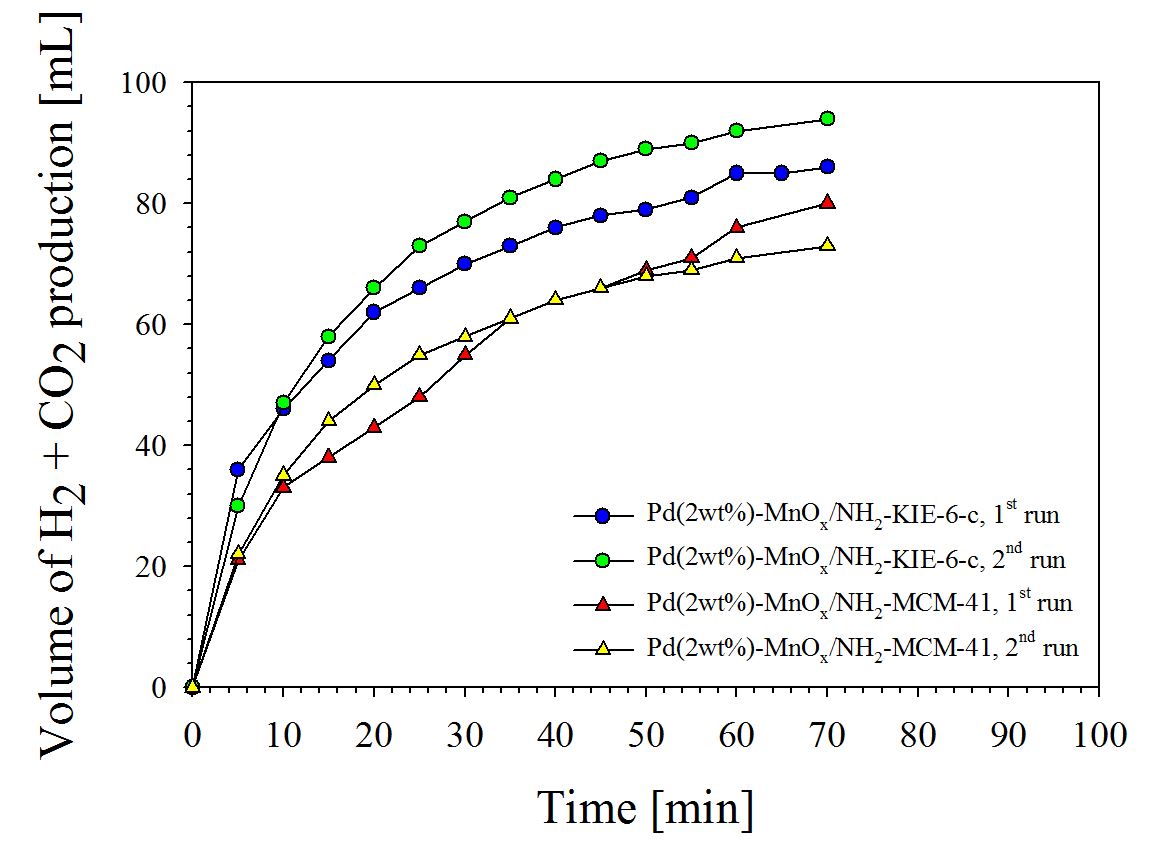
**

**Figure S3. Recyclability test results of Pd(2wt%)-MnO_x_/NH_2_-KIE-6-c and Pd(2wt%)-MnO_x_/NH_2_-MCM-41.**


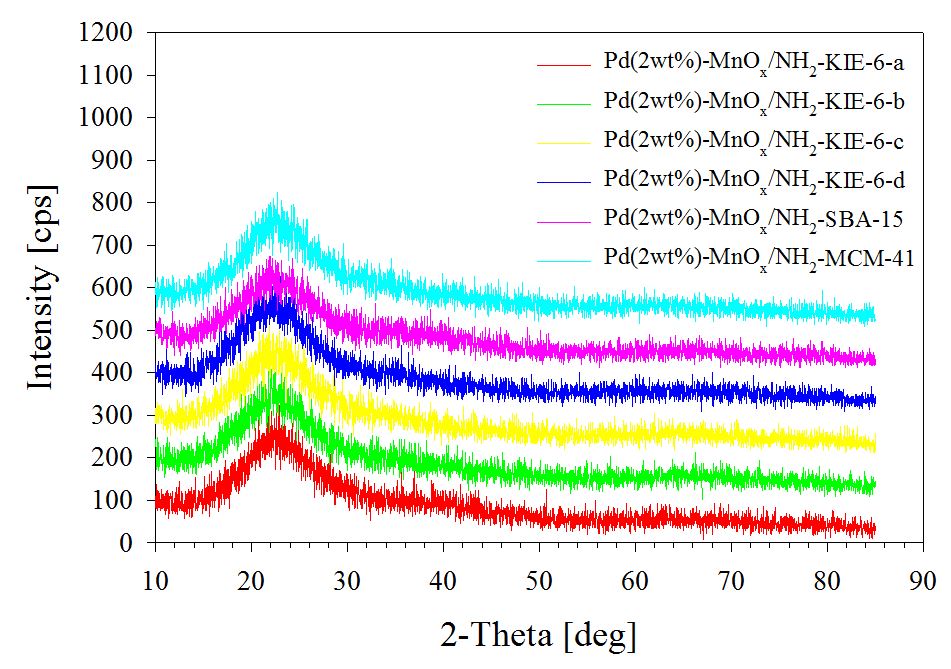


**Figure S4. XRD patterns of Pd(2wt%)-MnO_x_(Mn basis 4 wt%)/ KIE-6-a, Pd(2wt%)-MnO_x_(Mn basis 4 wt%)/ KIE-6-b, Pd(2wt%)-MnO_x_(Mn basis 4 wt%)/ KIE -6-c, Pd(2wt%)-MnO_x_(Mn basis 4 wt%)/ KIE-6-d, Pd(2wt%)-MnO_x_(Mn basis 4 wt%)/SBA-15 and Pd(2wt%)-MnO_x_(Mn basis 4 wt%)/MCM-41.**


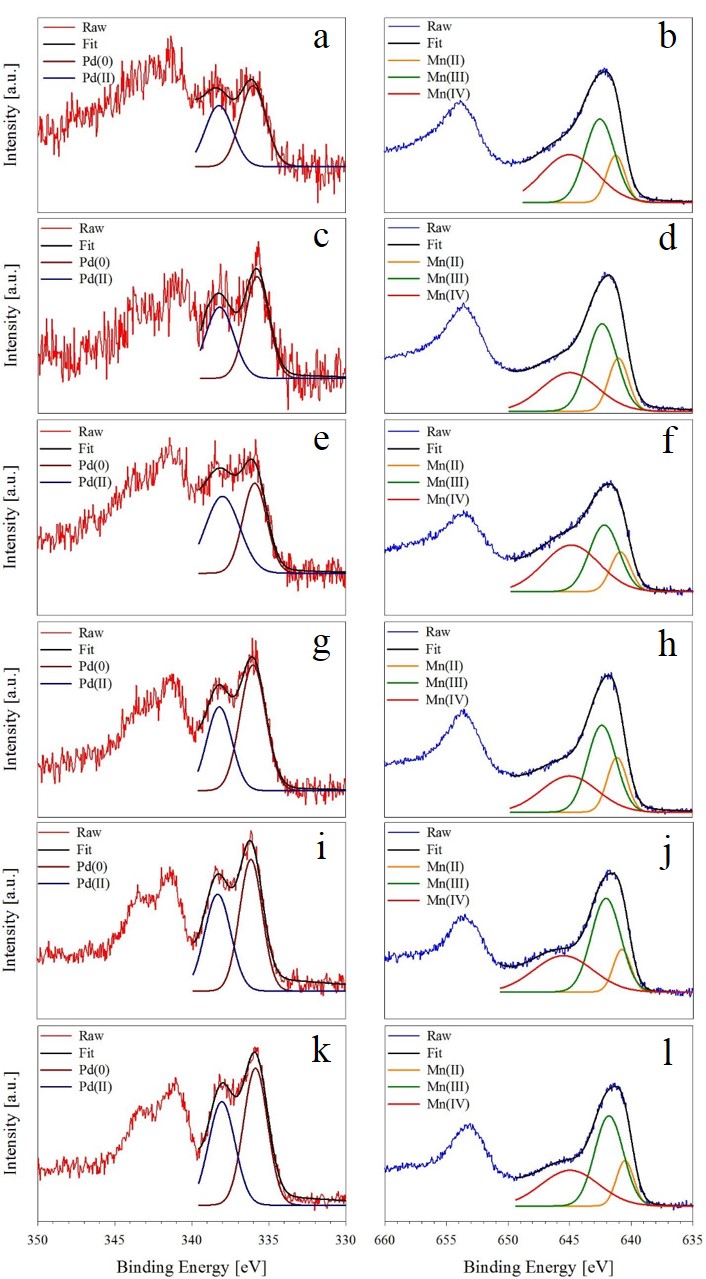


**Figure S5. Deconvoluted XPS spectra of catalysts.** (a,b) Pd(2wt%)-MnO_x_(Mn basis 4 wt%)/ KIE-6-a, (c,d) Pd(2wt%)-MnO_x_(Mn basis 4 wt%)/ KIE-6-b, (e,f) Pd(2wt%)-MnO_x_(Mn basis 4 wt%)/ KIE -6-c, (g,h) Pd(2wt%)-MnO_x_(Mn basis 4 wt%)/ KIE-6-d, (i,j) Pd(2wt%)-MnO_x_(Mn basis 4 wt%)/SBA-15, (k,l) Pd(2wt%)-MnO_x_(Mn basis 4 wt%)/MCM-41. (a,c,e,g,i,k) Pd 3d spectra. (b,d,f,h,j,l) Mn 2p spectra.


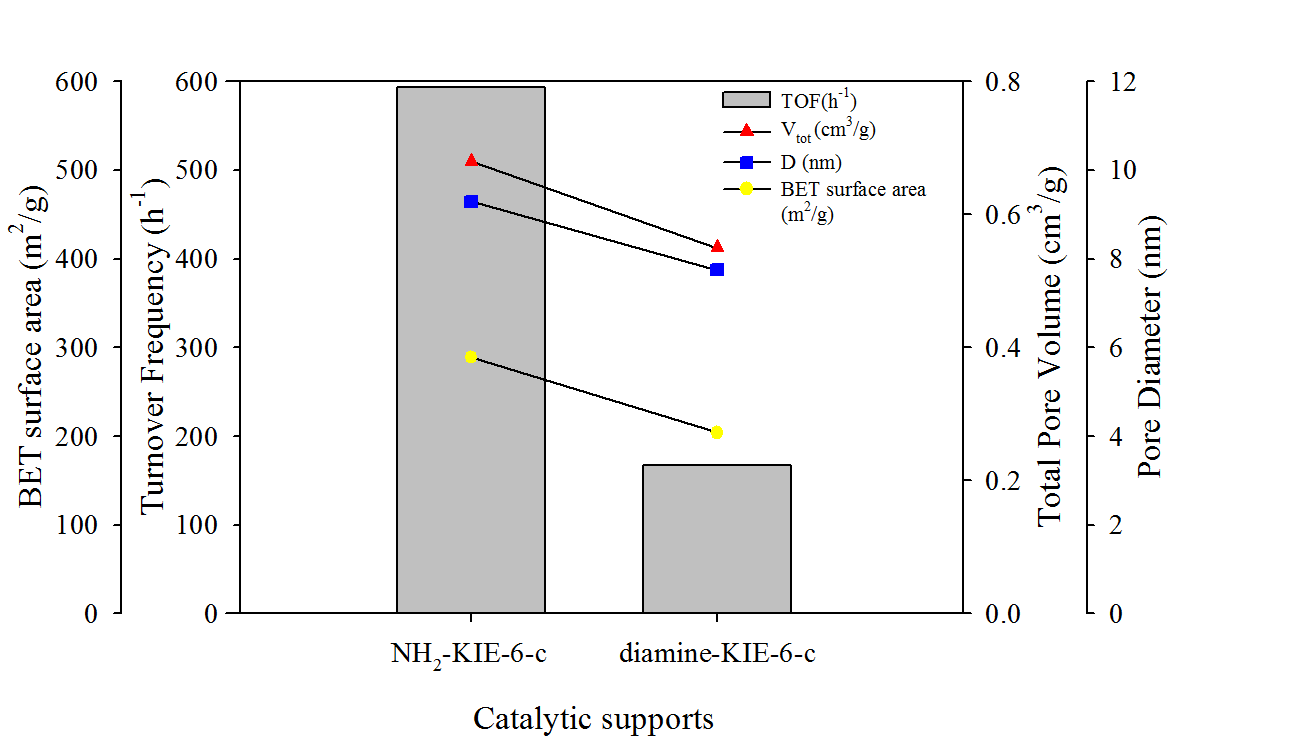


**Figure S6. Correlation between TOF and pore properties of catalytic supports.** (TOF values are for Pd(2wt%)-MnO_x_(Mn basis 4 wt%)/NH_2_-KIE-6-c or /diamine-KIE-6-c.)
